# Cellular effects of fluorodeoxyglucose: Global changes in the lipidome and alteration in intracellular transport

**DOI:** 10.18632/oncotarget.13089

**Published:** 2016-11-04

**Authors:** Simona Kavaliauskiene, Maria Lyngaas Torgersen, Anne Berit Dyve Lingelem, Tove Irene Klokk, Tuulia Lintonen, Helena Simolin, Kim Ekroos, Tore Skotland, Kirsten Sandvig

**Affiliations:** ^1^ Department of Molecular Cell Biology, Institute for Cancer Research, Oslo University Hospital, Oslo, Norway; ^2^ Center for Cancer Biomedicine, Oslo University Hospital, Oslo, Norway; ^3^ Department of Biosciences, University of Oslo, Oslo, Norway; ^4^ Zora Biosciences, Espoo, Finland

**Keywords:** Shiga toxin, glucosylceramide, lipidomics, intracellular transport, 2-fluoro-2-deoxy-D-glucose

## Abstract

2-fluoro-2-deoxy-D-glucose (FDG), labeled with ^18^F radioisotope, is the most common imaging agent used for positron emission tomography (PET) in oncology. However, little is known about the cellular effects of FDG. Another glucose analogue, 2-deoxy-D-glucose (2DG), has been shown to affect many cellular functions, including intracellular transport and lipid metabolism, and has been found to improve the efficacy of cancer chemotherapeutic agents *in vivo*. Thus, in the present study, we have investigated cellular effects of FDG with the focus on changes in cellular lipids and intracellular transport. By quantifying more than 200 lipids from 17 different lipid classes in HEp-2 cells and by analyzing glycosphingolipids from MCF-7, HT-29 and HBMEC cells, we have discovered that FDG treatment inhibits glucosylceramide synthesis and thus reduces cellular levels of glycosphingolipids. In addition, in HEp-2 cells the levels and/or species composition of other lipid classes, namely diacylglycerols, phosphatidic acids and phosphatidylinositols, were found to change upon treatment with FDG. Furthermore, we show here that FDG inhibits retrograde Shiga toxin transport and is much more efficient in protecting cells against the toxin than 2DG. In summary, our data reveal novel effects of FDG on cellular transport and glycosphingolipid metabolism, which suggest a potential clinical application of FDG as an adjuvant for cancer chemotherapy.

## INTRODUCTION

2-fluoro-2-deoxy-D-glucose (FDG) is a structural analogue of glucose where the hydroxyl group at the C-2 position is replaced by a fluorine atom. [^18^F]FDG, with its incorporated ^18^F radioisotope, is the most common imaging agent used for positron emission tomography (PET) in the clinic. [^18^F]FDG-PET has been established as a standard technique for staging and monitoring of multiple cancers (for review see [1]). The use of [^18^F]FDG-PET in oncology is based on an increased uptake and metabolism of glucose in cancer cells, which leads to higher accumulation of [^18^F]FDG in tumors compared to surrounding tissues.

Like glucose, FDG is transported into cells, where it is phosphorylated by hexokinase to yield FDG-6-P. However, FDG-6-P does not undergo isomerization to fructose and thus cannot be further catabolised, leading to the accumulation of FDG-6-P in the cells [2]. Similarly to 2-deoxy-D-glucose (2DG), a commonly used glycolytic inhibitor, FDG also inhibits glycolysis by (i) competing with glucose-6-P for phosphoglucose isomerase, and by (ii) acting as a non-competitive inhibitor of hexokinase [3–5]. The binding energy of FDG-6-P for the allosteric site of the hexokinase is lower than that of 2DG-6-P, and closely resembles the energy of glucose-6-P, making it a better inhibitor of glycolysis than 2DG [5]. As a consequence, FDG is more efficient than 2DG in killing hypoxic cells [5].

In addition to the inhibition of glycolysis, both 2DG and FDG inhibit N-linked protein glycosylation [6–8]. Protein N-glycosylation involves the assembly of an oligosaccharide on a lipid carrier, dolichol pyrophosphate, and the transfer of the oligosaccharide onto an acceptor protein. Although FDG has been found to be converted to GDP-FDG and UDP-FDG in cells [8], it does not compete with UDP-GlcNAc or GDP-mannose for addition onto dolichol-linked oligosaccharides [7]. FDG has been suggested to interfere with N-glycosylation by (i) competing with mannose and glucose for the formation of GDP-mannose and UDP-glucose, and (ii) the nucleotide diphosphate-linked FDG is suggested to inhibit the addition of carbohydrates from GDP-mannose and UDP-glucose onto dolichol, leading to slower assembly of the dolichol-linked oligosaccharides [2, 7]. In contrast, 2DG has been shown to become incorporated into dolichol-linked oligosaccharides, which results in termination of the oligosaccharide elongation, and thus inhibits the transfer of the shortened oligosaccharides onto the proteins [6], making 2DG a better inhibitor of N-glycosylation than FDG. 2DG has been extensively studied since the 1960s, and has been revealed to interfere with cell cycle control [9] and DNA repair [10], to induce autophagy [11] and apoptosis [12], and to modify cellular lipid composition and intracellular trafficking [13], remarkably not always by the mechanisms dependent on the inhibition of glycolysis or N-glycosylation [13, 14]. Surprisingly, it has not been studied whether FDG also affects any of these processes.

We have recently reported that cell treatment with 2DG leads to multiple changes in cell lipid composition, affecting both the levels and species composition (the saturation and length of fatty acyl groups) of several lipid classes, and also leads to 2DG incorporation into the carbohydrate moiety of glycosphingolipids (GSLs) [13]. Importantly, not only proteins, but also lipids are involved in cellular signaling and in controlling many cellular processes. For instance, changes in GSLs have been shown to alter cell proliferation, autophagy, apoptosis, endocytosis, intracellular transport, migration, senescence and inflammation (for review see [15] and [16]), all of these processes being crucial in tumorigenesis, cancer progression and response to treatment [16, 17]. In addition, the over-expression of glucosylceramide synthase (GCS), the enzyme catalyzing the first reaction of ceramide (Cer) glycosylation, has been associated with drug-resistance and poor response to chemotherapy in a variety of cancers (for review see [18]).

Protein toxins have proven useful as tools to investigate changes in cellular processes, such as endocytosis, intracellular transport and sorting (for review see [19]). Plant and bacterial toxins bind to a large variety of cell surface receptors. For instance, Shiga toxins (Stx), produced by *Shigella dysenteriae* and strains of *Escherichia coli*, bind exclusively to globotriaosylceramide (Gb3), while the plant toxin ricin binds to a variety of cell surface molecules, both glycoproteins and glycolipids with terminal galactose. For the cytotoxic action, receptor-bound Stx and ricin need to be endocytosed and transported retrogradely to the ER, where their enzymatically active moiety is translocated into the cytosol and inhibits protein synthesis (for review see [19]). Although Stx and ricin follow similar routes in the cell, their transport differs mechanistically, as ricin seems to explore a larger variety of pathways than Stx (for review see [20]). On the other hand, diphtheria toxin (DT), which binds to the heparin-binding epidermal growth factor precursor (pro-HB-EGF) [21], is directly translocated from endosomes into the cytosol upon low pH-induced conformational change [22], and thus does not require transport to ER for its cytotoxic action.

We have previously reported that the glucose analogue 2DG protects cells against Stx by inhibiting release of its cytotoxic A_1_-moiety in the ER via a mechanism that is not directly caused by the inhibition of glycolysis or N-glycosylation, but rather mediated via depletion of the ER calcium reservoirs [13]. In addition, 2DG treatment was found to change the cellular lipid composition, and 2DG became incorporated into the carbohydrate moiety of GSLs [13]. Thus, we have here investigated if FDG might have similar effects as 2DG on cells. Surprisingly, we found FDG to be 10-fold more effective than 2DG in protecting cells against Stx, and to inhibit Stx transport from the Golgi to the ER via a yet unknown mechanism. In addition, our quantitative mass spectrometry (MS) lipid analyses show that FDG does not become incorporated into GSLs, but blocks glucosylceramide (GlcCer) biosynthesis and reduces the levels of GlcCer, lactosylceramide (LacCer) and Gb3. In summary, our data indicate that FDG treatment leads to multiple cellular changes both in intracellular transport and cellular lipids, suggesting that FDG might have yet overlooked therapeutic potential in addition to its established role as an imaging agent in PET.

## RESULTS

### FDG protects cells against Shiga toxins

**Figure 1:**
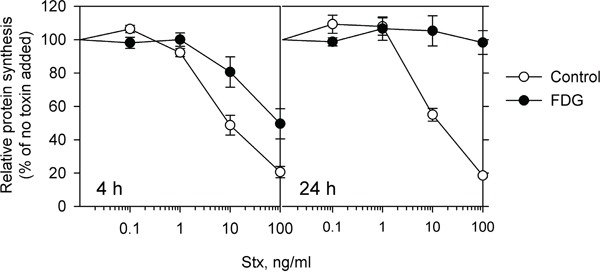
FDG protects cells against Stx Cells were incubated with or without 1 mM FDG for 4 and 24 h followed by incubation with 10-fold serial dilutions of Stx for 3 h in the presence or absence of FDG, and protein synthesis was measured. The graph shows mean ± SEM from four independent experiments.

We have previously reported a novel effect of the glucose analogue 2DG on the intracellular transport of Stx. Treatment with 2DG impairs Stx release from the ER and thus protects cells against the toxin [13]. To further investigate the potential effects of glucose analogues on intracellular transport, we have here tested the related glucose analogue, FDG. Surprisingly, we found FDG to be ten-fold more effective than 2DG in protecting HEp-2 cells against Stx. Pretreatment of cells with 1 mM FDG for 4 h increased the IC_50_ (the concentration of the toxin required to result in 50% reduction in protein synthesis) by 13-fold (Figure 1 and Supplementary Figure S1), while a ten times higher concentration of 2DG (10 mM) was required to give a similar protection against Stx [13]. In addition, 24 h treatment with 1 mM FDG made HEp-2 cells fully resistant to Stx (Figure 1). Intriguingly, when the cell medium was replaced with fresh complete growth medium after 4 h or 24 h pretreatment with 1 mM FDG, cell sensitivity to Stx was only partially restored after 24 h and 48 h (Supplementary Figure S1C), which was not the case for 2DG, as a 24 h wash-out fully restored the sensitivity to Stx (data not shown). This indicates that FDG induces long-lasting cellular changes different from those mediated by 2DG.

Proteins toxins have different requirements for binding to cells and intracellular transport, and we have therefore also tested whether FDG provides a protective effect against other protein toxins, such as Shiga-like toxin 2 (Stx2), ricin and diphtheria toxin (DT). In line with data for 2DG [13], FDG had no protective effect against DT, and only a weak (approx. 2-fold) protection against ricin following even 24 h preincubation with 1 mM FDG (Supplementary Figure S1B). Importantly, the protective effect against Stx2, which is transported to the Golgi apparatus by a different mechanism than Stx [23] and is shown to be more potent than Stx *in vivo* [24, 25], was similar to that observed for Stx (Supplementary Figure S1B).

It should be mentioned that 1 mM FDG, the concentration chosen to be tested in all further experiments, induced only a slight reduction in HEp-2 cell proliferation (appr. 10% after 48 h), and had a minor inhibitory effect (approx. 15%) on protein synthesis (Supplementary Figure S2), indicating that FDG treatment did not lead to significant inhibition of glycolysis and ATP depletion at the conditions used. In addition to the inhibition of glycolysis, FDG also interferes with protein N-glycosylation [6, 7]. However, combined treatment with mannose, which rescues protein N-glycosylation [6], did not rescue cell sensitivity to Stx (Supplementary Figure S3), indicating that the protection is not mediated via aberrant protein N-glycosylation.

Finally, to test whether FDG-induced protection against Stx is limited to HEp-2 cells only, we analyzed Stx toxicity in three additional cell lines: MCF-7 (human breast adenocarcinoma), HT-29 (human colorectal adenocarcinoma) and HBMEC (transformed human brain microvascular endothelial cells). Both 4 h and 24 h pretreatment with 1 mM FDG reduced HT-29 and HBMEC cell sensitivity to Stx (Supplementary Figure S4). MCF-7 cells are much less sensitive to Stx, which makes it difficult to draw conclusions from the toxicity data on these cells, but FDG seems to reduce MCF-7 cell sensitivity to Stx as well (Supplementary Figure S4).

### FDG inhibits Stx binding and endocytosis

**Figure 2:**
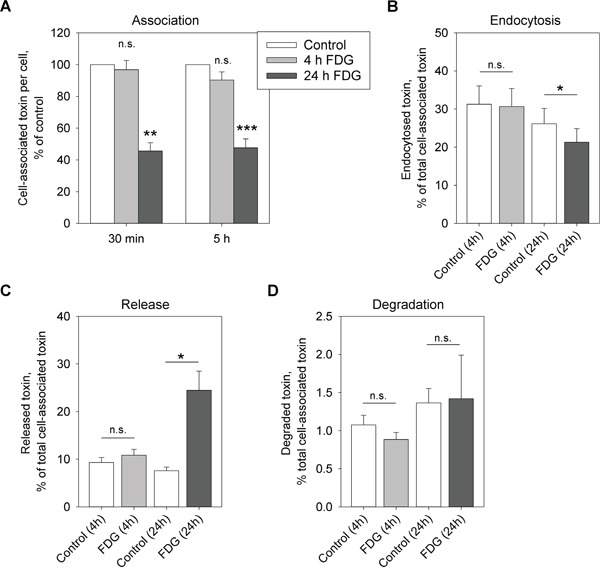
FDG reduces Stx binding and endocytosis, and leads to increased release of the toxin back to the medium Cells were treated with 1 mM FDG for 4 or 24 h. **A.** ^125^I-Stx1-mut was added and the incubation was continued for 30 min or 5 h. Cell-associated toxin was measured and normalized to cell number. **B.** Cells were incubated with ^125^I-Stx1-mut-biotin for 20 min, the endocytosed ^125^I-Stx1-mut-biotin was quantified in cell lysates and normalized to the total cell-associated toxin. **C** and **D.** Cells were incubated with ^125^I-Stx1-mut for 30 min, the non-bound toxin was washed away and the cells were incubated with fresh medium for 1 h. The released and degraded toxin was determined as described in Materials and Methods. (C) Shows released and (D) shows degraded ^125^I-Stx1-mut as a percentage of total cell-associated toxin. All figures show mean values + SEM from at least three independent experiments; one-sample Student's t-test was used for (A) and paired Student's t-test was used for (B-D), *p<0.05, **p<0.005, ***p<0.0005.

For its cytotoxic action, Stx needs to bind Gb3, become endocytosed and be sorted along the retrograde pathway to the ER where its enzymatically active A_1_-subunit is released into the cytosol and inhibits proteins synthesis. Interfering with any of these steps would lead to cell protection against Stx. Therefore, we first investigated if FDG had any effect on Stx association with the cells. Indeed, 24 h treatment with FDG followed by 30 min or 5 h incubation with Stx1-mut (non-toxic Stx1 mutant), led to 54% and 52% reduction, respectively, in toxin association with HEp-2 cells (Figure 2A). However, there was no effect on Stx binding following 4 h treatment (Figure 2A), although, 4 h preincubation is sufficient to provide a 13-fold protection (Figure 1 and Supplementary Figure S1). In addition, when Stx endocytosis was analyzed, it was only 24 h, and not 4 h, treatment that gave a significant reduction in Stx endocytosis (Figure 2B). Moreover, we analyzed the release of Stx back to the medium once it has been bound to the cells, and we observed a significant increase in Stx release following 24 h, but not 4 h, treatment with FDG (Figure 2C). The degradation of Stx was not affected by FDG (Figure 2D), suggesting that the increase in Stx release after 24 h treatment is due to increased Stx recycling and/or release from the receptor.

### FDG treatment reduces GlcCer, LacCer and Gb3, and changes cellular lipid composition in HEp-2 cells

Stx binding and intracellular transport has been shown to be modulated by the Gb3 composition (different Gb3 species have been shown to be required for efficient binding [26–28]), as well as by the membrane environment of the receptor [26, 29]. Therefore, to investigate the mechanism by which FDG inhibits Stx binding, we performed lipidomic analyses of HEp-2 cells following 4 h and 24 h treatment with FDG. In total, 230 lipid species from 17 lipid classes were quantified (the full list and values of the quantified lipid species are given in Supplementary Table S1).

**Figure 3:**
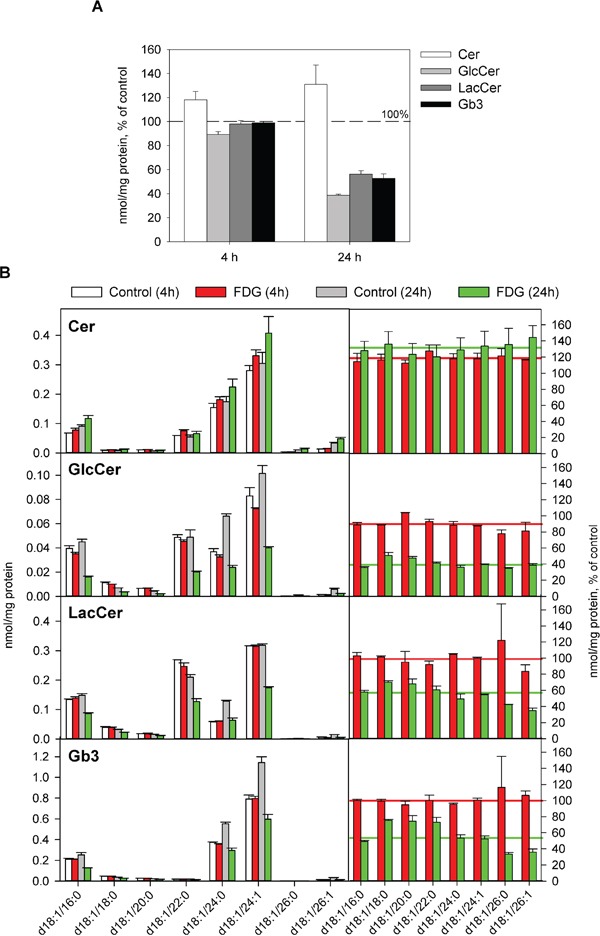
Effect of FDG treatment on total levels and species composition of Gb3 and its precursors Cells were treated with or without 1 mM FDG for 4 or 24 h, and whole-cell lysates were analysed by MS. **A.** The levels of Cer, GlcCer, LacCer and Gb3 in FDG treated cells compared to control samples; the error bars show the deviation from the mean of two biological samples. **B.** Species composition of Cer, GlcCer, LacCer and Gb3 in control and FDG treated samples. Right panels show relative amount of each species compared to the levels in the controls. Red and green lines depict the levels of the total class after 4 and 24 h treatment with FDG, respectively.

We have recently shown that 24 h treatment with 10 mM 2DG leads to approximately 50% reduction in total Gb3 and accumulation of LacCer in the cells upon longer incubations [13]. Here we found that 24 h treatment with 1 mM FDG gave a similar reduction in total Gb3, but in contrast to the results obtained with 2DG, FDG treatment also reduced the cellular levels of LacCer and GlcCer (Figure 3A). Importantly, a slight reduction in total GlcCer was observed already after 4 h, and the levels of total Cer were increased in both 4 h and 24 h treated samples (Figure 3A), indicating that FDG blocks the synthesis of GlcCer. FDG treatment did not change the composition of the major species of GlcCer, LacCer and Gb3 (those containing the N-amidated fatty acyl group with C16:0, C24:0 or C24:1), as they were reduced to the same level as the whole class (Figure 3B). The relative increase in species with fatty acyl chains containing 18, 20 and 22 carbon atoms and the decrease in the two species with 26 carbon atoms should be interpreted with care due to very low abundance of these species (Figure 3B).

The reduction in Gb3 upon 2DG treatment is mediated via transcriptional down-regulation of the Gb3 synthase (α-1,4-galactosyltransferase), which transfers galactose to LacCer to form Gb3 [13, 30]. However, we did not observe a significant reduction in the mRNA levels of either glucosylceramide synthase (GCS) or Gb3 synthase following 6 h treatment with FDG (Supplementary Figure S5), suggesting that it is not an inhibited expression of the enzymes that leads to reduction in the GSLs in response to FDG treatment.

**Figure 4:**
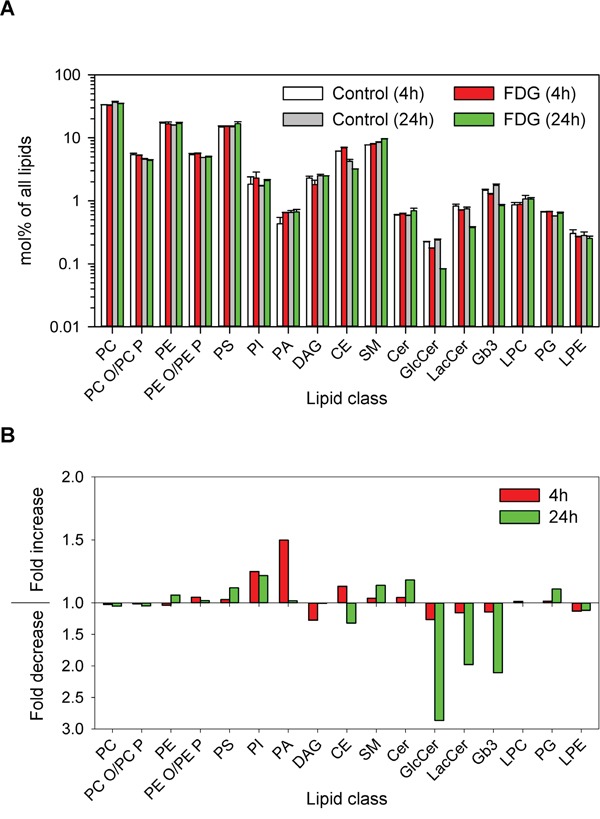
Changes in lipid composition after FDG treatment Cells were treated with or without 1 mM FDG for 4 or 24 h, and whole-cell lysates were analysed by MS. **A.** The relative amount of different lipid classes in the samples; the error bars show the deviation from the mean of two biological samples. **B.** Relative fold change in lipid classes after 4 and 24 h treatment with FDG compared to control samples.

The FDG-mediated effects on the cellular lipids were not limited to Gb3 and its precursors, as several other lipid classes were also affected by the treatment. We observed a 1.5-fold increase in total PA and a 1.2-fold increase in total PI after 4 h treatment with FDG, while there was an opposite effect on total DAG with a 1.2 fold decrease (Figure 4). After 24 h, there was still an increase in total PI (1.2-fold), whereas no changes in total PA or DAG were observed when compared with the control samples (Figure 4). Interestingly, the species composition of these three lipid classes was also changed in response to FDG treatment (Supplementary Figure S6). There was a reduction in PA 16:1_18:1 and an increase in PA 18:1/18:1 (both in absolute values and as mol% of the total class) after 4 h and 24 h treatment. For the DAG, the main change was a reduction in DAG 16:1_18:1, which was also responsible for more than half of the reduction in total DAG after 4 h treatment. The composition of PI also changed with an increase in mol% of PI 18:0_18:1 and a decrease in PI 18:1/18:1 after both incubation times. Interestingly, FDG-mediated changes in lipid composition were different from those reported for 2DG [13]. For instance, FDG had no effect on the levels of the lysophospholipids LPC and LPE, which were reduced by up to 1.5 fold upon 2DG treatment. In addition, although both FDG and 2DG affected DAG, PA and PI levels, the effects are clearly different both on the total levels and the species composition of these lipids. However, it is important to note that 2DG treatment inhibits cell growth, and that some of the changes reported for 2DG can be associated with reduced cell growth [31]. It can therefore be a challenge to directly compare the 2DG- and FDG-mediated effects.

We have previously reported [13] that 2DG becomes incorporated into newly synthesized Gb3 and its precursors (approx. 20% GlcCer and LacCer contained 2DG after 24 h incubation); therefore we analyzed if FDG could also become incorporated into GSLs. However, based on our lipidomic analysis, we did not observe any incorporation of FDG into Gb3 or its precursors (data not shown). The lack of FDG incorporation into GSLs is either caused by the inhibition of the GlcCer synthesis as such, or due to that GlcCer synthase discriminates between UDP-FDG and UDP-glucose and does not transfer FDG onto Cer.

Finally, we analyzed whether Gb3 levels are restored in HEp-2 cells when FDG is removed from the medium. HEp-2 cells were either continuously treated with 1 mM FDG for 24 h or 72 h, or following 24 h pretreatment with FDG the cells were washed with fresh medium and grown in complete growth medium without the drug for 48 h. Cellular GSLs were extracted from control and treated samples as described in the Supplementary Methods and then analyzed by high performance thin layer chromatography (HPTLC). Compared to control cells, cellular content of GlcCer, LacCer and Gb3 was reduced down to 35%, 40% and 45%, respectively, in the cells treated with 1 mM FDG for 24 h (Supplementary Figure S7). Importantly, when following 24 h treatment with FDG cells were grown in fresh medium for 48 h prior to lipid extraction and HPTLC analysis, cellular content of GlcCer was fully restored to control levels, and the levels of LacCer and Gb3 were partially restored, to approx. 60% and 80%, respectively, to that of control (Supplementary Figure S7). GlcCer has a shorter turnover time than LacCer or Gb3 in HEp-2 cells [32], which might explain why GlcCer levels are fully restored within 48 h, while LacCer and Gb3 remains still lower than in control.

### FDG reduces glycosphingolipid levels and Stx binding in MCF-7, HT-29 and HBMEC cells

To investigate whether FDG-induced changes in cellular lipids, in particular reduction in Gb3 and its precursors, are restricted to HEp-2 cells, we performed HPTLC analysis of GSLs extracted from MCF-7, HT-29 and HBMEC cells treated with or without 1 mM FDG for 24 h or 72 h. Already after 24 h treatment there was a clear reduction (more than 40%) in cellular levels of Gb3 and its precursors in all the cell lines tested, and there was an additional reduction in GSL levels following 72 h treatment with FDG (Supplementary Figure S8). In addition, we analyzed whether Stx binding was also reduced in these cells upon FDG treatment, as there was a significant reduction in Gb3 levels in all the cells after FDG treatment. We found that both 24 h and 72 h treatment with 1 mM FDG reduced Stx binding on ice to all the cells tested, although to different extent (Supplementary Figure S9). After 72 h treatment, Stx binding to MCF-7 and HT-29 cells was completely lost, while it was reduced by approx. 60% and 30% in HEp-2 and HBMEC cells, respectively (Supplementary Figure S9). Thus the overall inhibitory effect of FDG on GSL synthesis and Stx binding seems to be universal and not cell type dependent, while the extent of the changes depends on the cell type.

### FDG inhibits Stx transport from the Golgi to the ER

While the reduction in Gb3 levels and inhibition of Stx binding might partially explain the protection against Stx following 24 h treatment with FDG, it does not explain the protection against the toxin following 4 h treatment. We therefore studied which effects FDG has on Stx intracellular transport following 4 h treatment, a time point when there are no significant effects on the Gb3 levels and Stx binding.

**Figure 5:**
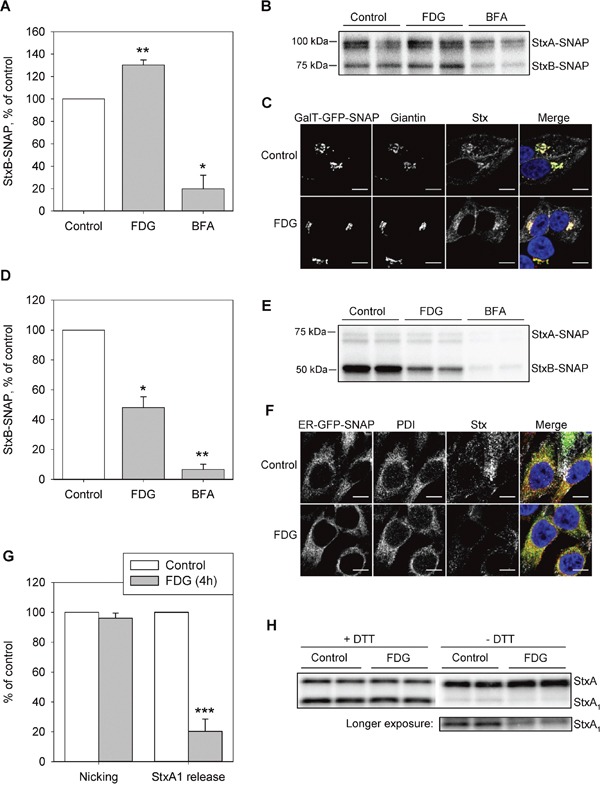
FDG inhibits Stx transport to the ER and the release of StxA_1_ **A** and **B.** HEp-2-GalT-GFP-SNAP cells were treated with 1 mM FDG for 4 h, or with 2 μg/ml BFA for 30 min prior to incubation with ^125^I-Stx1-mut-BG for 1 h in the presence of the drugs. Cells were lysed, and Stx-SNAP was immunoprecipitated and run on SDS-PAGE. (A) The quantification of StxBbound to SNAP-tag. A representative autoradiogram is shown in (B). **C.** HEp-2-GalT-GFP-SNAP cells were treated with FDG as in (A) and (B) prior to incubation with Stx1-mut-BG-Alexa555. The cells were fixed, permeabilized and immunolabeled for giantin, prior to mounting with Prolong®Gold with DAPI, and imaged. The overlay image shows GalT-GFP-SNAP signal in green, giantin in red, Stx in grey and DAPI in blue. Scale bar, 10 μm. **D** and **E.** HEp2-ER-GFP-SNAP cells were treated as in (A) and (B) prior to incubation with ^125^I-Stx1-mut-BG for 5 h. Cells were lysed and Stx-SNAP was immunoprecipitated and run on SDS-PAGE. (D) The quantification of StxB bound to SNAP-tag. A representative autoradiogram is shown in (E). **F.** HEp-2-ER-GFP-SNAP cells were treated with FDG as in (A) and (B), and incubated with Stx1-mut-BG-Alexa555 for 1 h, followed by 4 h incubation with fresh medium. Cells were fixed, permeabilized and immunolabeled for PDI, prior to mounting with Prolong®Gold with DAPI, and imaged. The overlay image shows ER-GFP-SNAP signal in green, PDI in red, Stx in grey and DAPI in blue. Scale bar, 10 μm. **G** and **H.** HEp-2 cells were treated with 1 mM FDG for 4 h prior to incubation with ^125^I-Stx1-mut for 5 h at 37°C. Cell lysates were separated by reducing (+DTT) or non-reducing (−DTT) SDS-PAGE to determine the nicking of StxA or StxA_1_ release, respectively. A representative autoradiogram is shown in (H). The amount of released or nicked StxA_1_ was calculated as a percentage of total Stx (the sum of StxA and StxA_1_). (A), (D) and (G) show mean +SEM from at least three independent experiments; *p<0.05, **p<0.01, *** p<0.001, one sample Student's *t*-test.

We analyzed three subsequent steps of the intracellular Stx transport: (i) transport to the Golgi, (ii) transport from the Golgi to the ER, and (iii) release of the enzymatically active StxA_1_ moiety in the ER. To study steps (i) and (ii) we adapted a recently developed strategy for analyzing retrograde protein transport [33] which is based on a SNAP-tag® technology [34]. The SNAP-tag enzyme reacts specifically and rapidly with benzylguanine (BG) derivatives, leading to irreversible covalent labeling of the SNAP-tag with the probe attached to the BG molecule. We generated two stable HEp-2 cell lines expressing Golgi localized-SNAP (HEp-2-GalT-GFP-SNAP) or endoplasmic reticulum (ER) localized-SNAP (HEp-2-ER-GFP-SNAP). The correct cellular localization of the constructs was confirmed by confocal microscopy using giantin and PDI immunolabeling for marking the Golgi and the ER, respectively (Figure 5C and 5F).

Since BFA disrupts the Golgi and inhibits Stx transport [35, 36], cell treatment with 1 μg/ml BFA 30 min prior to addition of the toxin was used as a positive control for reduction in retrograde Stx transport. Surprisingly, when HEp-2-GalT-GFP-SNAP cells were treated with FDG for 4 h prior to 1 h incubation with ^125^I-Stx1-mut-BG, we observed a significant 30% increase in StxB-SNAP signal in the Golgi (Figure 5A), suggesting that FDG did not disrupt Stx transport to the Golgi. Next, by using HEp2-ER-GFP-SNAP cells, we tested Stx transport to the ER and found that 4 h pretreatment with FDG reduced Stx labeling with the SNAP-tag by 50% (Figure 5D). Taken together, the data suggest that FDG inhibits Stx transport from the Golgi to the ER.

Finally, we tested if the release of the StxA_1_from the holotoxin is affected by FDG, as 2DG has been shown to inhibit this step of the Stx transport [13]. StxA requires nicking by the protease furin to form A_1_- and A_2_-moieties, which are connected by a disulfide bond. The nicking occurs in the endosomes and/or the Golgi apparatus [37]. In the ER, reduction of the disulfide bond connecting the A_1_- and A_2_-moieties releases StxA_1_ from the holotoxin. To quantify the release of the StxA_1_ from the holotoxin, cells were incubated with ^125^I-Stx1-mut for 5 h followed by SDS-PAGE under reducing (+DTT) and non-reducing (−DTT) conditions. In the autoradiogram, the bottom band represents StxA_1_ (27 kDa) and the top band represents StxA (32 kDa) (Figure 5H). There was no reduction in the proteolytically cleaved Stx following 4 h treatment with FDG (Figure 5G; Nicking), but there was an 80% reduction in the reductive release of StxA_1_ (Figure 5G; StxA1 release). Importantly, the FDG-induced reduction in StxA_1_ release from the holotoxin is more pronounced than the reduction in Stx transport to the ER (p=0.046, two-sample unequal variance Student's t-test comparing Stx-SNAP(ER) and StxA_1_ in FDG samples), suggesting that FDG might have an additional effect on StxA_1_ release from the holotoxin in the ER.

We have previously shown that the inhibitory effect of 2DG on the release of StxA_1_ might be mediated via depletion of ER calcium stores upon 2DG treatment [13]. To test whether FDG also depletes Ca^2+^ from the ER, we used Fluo-4 NW calcium assay to measure whether pretreatment with FDG would alter thapsigargin (TG) induced Ca^2+^ release from the ER. TG inhibits the ER Ca^2+^ ATPase and thus results in a rapid Ca^2+^ release from the ER. 2DG was used as a positive control, and 4 h treatment with 10 mM 2DG completely diminished the increase in cytosolic calcium (a peak in the measured fluorescence) upon addition of TG (Supplementary Figure S10). Importantly, the increase in cytosolic calcium upon TG addition was also reduced in cells treated with 1 mM FDG for 4 h, suggesting that FDG partially depleted Ca^2+^ from the ER, even though to a lower extent than treatment with 10 mM 2DG (Supplementary Figure S10).

## DISCUSSION

Here we show for the first time that FDG treatment induces multiple effects on intracellular transport and cell lipid composition. FDG specifically depletes HEp-2 cells for GlcCer, LacCer and Gb3 without affecting SM, and also alters total levels and/or species composition of several other lipid classes, namely DAG, PA and PI. Furthermore, FDG protects HEp-2 cells against Stxs, both by down-regulating the receptor (Gb3), and by inhibiting Stx transport from the Golgi to the ER and, in the ER, inhibiting the release of the enzymatically active StxA_1_ moiety. Importantly, the protective effect against Stx and the down regulation of Gb3 are not limited to HEp-2 cells only, as similar effects were also observed in other cells. Although it takes some time before FDG exerts maximal protection against Stx, it might still be a candidate drug for the Stx-producing bacteria infections, since some protection is obtained even when FDG is added together with the toxin. Moreover, as described below, the lipid changes induced by FDG treatment makes it a candidate for adjuvant treatment for chemotherapy.

There has been two distinct transport pathways demonstrated for Cer transport from the ER to the Golgi, one mediated by the CERT protein and the other by transport vesicles. Cer transported via CERT is preferentially incorporated into SM rather than in GSLs [38], while the vesicular transport of Cer has been suggested to fuel the synthesis of GSLs in the Golgi. FDG might inhibit the synthesis of GlcCer in the Golgi by either a direct inhibition of GCS, or by inhibiting the vesicular transport of Cer from the ER to the Golgi. There are two known cellular effects of FDG, the inhibition of glycolysis and protein N-glycosylation, however, none of which seems to be involved in the inhibition of GSL biosynthesis. Under the conditions tested, FDG did not seem to deplete cellular ATP in HEp-2 cells, as (i) there was little effect on cell growth and protein synthesis, (ii) there was no vesiculation of the Golgi observed, which has been shown to occur in response to ATP depletion by 2DG [39], and (iii) the levels of SM were not depleted, although CERT-mediated Cer transport is shown to depend on ATP [38]. The inhibition of GlcCer synthesis by FDG does not seem to be mediated via the inhibition of N-glycosylation either, as 2DG, which is shown to be more efficient in inhibiting N-linked glycosylation than FDG [40], does not reduce cellular GlcCer levels [13]. It has been shown that FDG and 2DG are converted to UDP-FDG and UDP-2DG, respectively [8, 41]. However, in contrast to 2DG, which has been shown to become incorporated into GSLs [13], FDG does not become incorporated into GSLs, suggesting that UDP-FDG may accumulate in the cells and thus inhibit GlcCer synthesis via direct competition with UDP-Glc for GlcCer synthesis.

Intracellular transport is regulated not only by proteins, but also by lipids, thus changes in lipid composition might influence specific transport pathways. For example, both PA and DAG have been associated with retrograde protein transport from Golgi to the ER [42, 43]. DAG is thought to act as a scaffold by facilitating membrane curvature due to its conical shape, and also to recruit and activate proteins involved in membrane fission such as ADP-ribosylating factor GTPase-activating protein (ArfGAP1) [43]. In turn, ARF proteins are shown to activate PC-specific phospholipase D (PLD), which generates PA from PC [44]. As a result, PA has also been implicated in Golgi vesicle formation and budding [45]. Thus one may speculate whether FDG treatment might affect the activity of PA phosphatases (PAPs) or/and PLD leading to changed PA and DAG levels in the Golgi and thus result in inhibiting Stx transport from Golgi to the ER. This would also be in agreement with a recent study showing that the inhibition of LPP3 (a member of the PAP2 family that localizes to the Golgi and ER) impairs the transport of StxB from the Golgi to the ER [42]. In addition, it should be noted, that the inhibitory effect of DAG depletion on the retrograde transport depends on which pathway of the DAG production that is blocked. The most profound effect has been demonstrated after treatment with propanolol, which inhibits PAP and thus blocks DAG generation from PA, whereas a smaller effect was observed by the inhibitor U73122, which inhibits PI specific phospholipase C and thus blocks DAG generation from phosphoinositides PI4P and PI4,5P_2_ [43]. In contrast, no effect on retrograde protein transport was observed after treatment with Fumonisin B_1_, which inhibits ceramide synthase leading to lowered SM levels and reduced DAG generation from SM [43]. Thus it has been hypothesized that specific DAG species might be required for the retrograde transport. In agreement with this idea, we have previously shown that certain DAG species are up- and down-regulated in cells grown at high density, which renders the cells less sensitive to Stx [31]. However, one has to note that our lipidomic studies were performed on whole cell lysates and thus do not directly reflect lipid changes in the Golgi. Therefore, additional studies are required to reveal if FDG affects enzymes involved in lipid metabolism, and if this is the mechanism by which FDG mediates its effects on intracellular transport.

2DG has been shown to improve the efficacy of several cancer chemotherapeutic agents *in vivo* [46, 47], and has already been tested as an adjuvant for chemotherapy in clinical trials [48]. However, while both FDG and 2DG have been shown to kill hypoxic cells *in vitro*, only 2DG was also found to be toxic at aerobic conditions, suggesting that FDG might have fewer side effects that 2DG when used in the clinic [5]. In addition, FDG has recently been reported to be effective in selectively killing hypoxic cells and to decrease tumor burden in an *in vivo* transgenic model of retinoblastoma [49], indicating that non-toxic FDG doses may have significant cellular effects *in vivo*. The over-expression of GCS has been associated with drug-resistance and poor response to chemotherapy in a variety of cancers, and the inhibition of GlcCer synthesis has been shown to sensitize cancer cells to chemotherapy (for review see [18]). We show here that FDG treatment effectively inhibits GlcCer synthesis in HEp-2 cells and reduces glycosphingolipid levels in several different cancer cell lines tested, suggesting that FDG might potentially improve chemotherapy in drug-resistant tumors. Importantly, FDG preferentially accumulates in tumor cells *in vivo,* and is already used as a contrast agent for PET in clinic, making it a good candidate for an adjuvant treatment for chemotherapy. Although the concentrations of FDG used for PET are low, in the nanomolar range or lower, clinical study with 2DG [48] suggest that the concentration of FDG used in this *in vitro* study might be achievable in patients, if 2DG and FDG display similar pharmacokinetic and toxic properties. In conclusion, the present study reveals novel effects of FDG on cellular transport and GSL metabolism, and opens up for potential new clinical applications of FDG.

## MATERIALS AND METHODS

### Materials

Purified Shiga toxin (Stx) was a kind gift from Dr. J. E. Brown (USAMRIID, Fort Detrick, MD, USA) and Dr. J. Kozlov (Academy of Science of Russia, Moscow, Russia). A non-toxic Shiga toxin 1 mutant (Stx1-mut) [50] was purified as previously described [31] from the pSW09 plasmid, which was a kind gift from Dr. A. D. O'Brien (Uniformed Services University of the Health Sciences, Bethesda, MD, USA). Shiga toxin 2 (Stx2) was purified as previously described [51]. Ricin was purchased from Sigma-Aldrich and diphtheria toxin was from Connaught Laboratories. The plasmid GalT-GFP-SNAP [33] was a kind gift from Prof. Ludger Johannes (Institut Curie, Paris, France), and the plasmid encoding the ER signal sequence of hen lysozyme fused to mCherry and KDEL was obtained from Prof. Harald Stenmark (University of Oslo, Norway).

The following primary antibodies were used: rabbit anti-giantin (Covance Inc.), rabbit anti-PDI (Santa Cruz Biotechnology). Fluorophore-conjugated secondary antibodies were from Jackson ImmunoResearch.L-[3,4,5-^3^H(N)]leucine and Na^125^I were from PerkinElmer. Other chemicals used were from Sigma-Aldrich unless otherwise stated.

Glycosphingolipids extracted from human erythrocytes was a kind gift from Prof. Dr. Johannes Müthing (Institute for medical Physics and Biophysics, University of Münster, Germany). HPTLC (high-performance thin-layer chromatography) silica gel 60 glass plates were purchased from Merck Millipore (Germany). For activation, the plates were heated for 30 min at 110°C, and cooled down before use.

### Cells

HEp-2 cells (ATCC/LGC, CCL-23) were cultured in DMEM+GlutaMAX™ (Gibco) medium supplemented with 10% (v/v) fetal bovine serum (FBS), 100 U/ml penicillin and 100 μg/ml streptomycin. HT-29 cells (purchased from ATCC in 2014) were cultured in DMEM/F12+GlutaMAX™ (Gibco) medium supplemented with 10% (v/v) FBS, 100 U/ml penicillin and 100 μg/ml streptomycin. MCF-7 cells (ATCC, ID test 100%, 2013) were cultured in RPMI 1640 medium supplemented with 10% (v/v) FBS, 100 U/ml penicillin and 100 μg/ml streptomycin. HBMEC (human brain microvascular endothelial cells) were a kind gift from Prof. J. Müthing (Institute of Hygiene, Münster, Germany), and were cultured in RPMI 1640 medium supplemented with 10% (v/v) FBS, 10% (v/v) Nu-serum (Corning), 2 mM L-glutamine, 1 mM sodium pyruvate, 1% (v/v) non-essential amino acids (NEAA), 1% MEM vitamin, 100 U/ml penicillin and 100 μg/ml streptomycin. If not otherwise specified, the culture medium components were purchased from Sigma-Aldrich.

Cells were grown in a humidified 5% CO_2_ atmosphere at 37°C. Unless specified otherwise, cells were seeded one day prior to the experiment, and the drugs were added directly to complete growth medium.

### Toxicity assay

Following the treatment with drugs, cells were washed with leucine-free HEPES buffered medium and incubated with ten-fold serial dilutions of Stx, ricin or diphtheria toxin in HEPES buffered medium with or without drugs for 3 h at 37°C. The incorporation of [^3^H]leucine was measured as described in [13]. The cytotoxicity was determined as the concentration of the toxin which reduced protein synthesis by 50% (IC_50_). Protection was defined as fold increase in IC_50_ in treated compared to control samples.

### Shiga toxin nicking and StxA_1_ release from the holotoxin

Stx1-mut was ^125^I-labeled by the iodogen method with the IODO-GEN Iodination Reagent (Pierce Biotechnology) as described previously [13].

After 4 h treatment with or without 1 mM FDG, 10 ng/ml ^125^I-Stx1-mut was added to the cells and the incubation was continued for 5 h at 37°C. Then, cells were washed with fresh HEPES buffered medium and incubated with 1 mM N-ethylmaleimide (NEM) in HEPES buffered medium for 5 min at 37°C. Finally, cells were lysed in sample buffer, and proteins were separated by non-reducing (−DTT) or reducing (+DTT) SDS-PAGE to determine the amount of released StxA_1_ or the amount of nicked StxA, respectively. Proteins were transferred to polyvinylidene difluoride (PVDF) membranes, visualized by autoradiography and quantified using the Quantity One 1-D Analysis Software (Bio-Rad Laboratories). The values were normalized to total StxA signal for each sample.

### Cell-association, release and degradation of Shiga toxin

To measure Stx association with cells, cells were treated with 1 mM FDG in complete growth medium prior to addition of the ^125^I-Stx1-mut (final conc. 20 ng/ml). The incubation with the toxin was continued for 30 min or 5 h at 37°C. The non-bound toxin was washed away and cell-associated toxin was measured in cell lysates by a γ-counter (1261 Multigamma Gamma Counter, Wallac).

To measure Stx release and degradation, the cells were incubated with 20 ng/ml ^125^I-Stx1-mut for 30 min at 37°C. The non-bound toxin was washed away, and the cells were incubated with fresh complete growth medium for 1 h at 37°C. The medium was collected, centrifuged at 400g for 5 min to remove any floating cells, and the radioactivity was counted in the supernatant (released Stx) and in the cell lysates (cell-associated Stx). To measure Stx degradation, the non-degraded ^125^I-Stx1-mut was precipitated from the medium by 5% (w/v) TCA, and the radioactivity in both degraded and nondegraded fractions was determined by a γ-counter. The released and degraded ^125^I-Stx1-mut was calculated as a percentage of total cell-associated toxin (cell associated + released toxin).

### Endocytosis of Shiga toxin

The endocytosis of Stx was quantified using a modified version of the procedure described previously [52]. The detailed description of the method is given in the Supplementary material 1. Briefly, Stx1-mut was biotinylated with the reducible EZ-Link Sulfo-NHS-SS-Biotin (Pierce Biotechnology) followed by the labeling with ^125^I (^125^I-Stx1-mut-biotin). After treatment with FDG, cells were incubated with 40 ng/ml ^125^I-Stx1-mut-biotin for 20 min at 37°C, and then half of the cells were treated with 0.1 M sodium 2-mercaptoethanesulfonate (MESNa), which reduces the SS-biotin from the cell surface-bound toxin. Cells were lysed, and ^25^I-Stx1-mut-biotin was captured by streptavidin-coated Dynabeads (Life Technologies). Endocytosis of Stx was calculated as internalized toxin (remaining signal after MESNa treatment) in percentage of total cell-associated toxin (mock treated cells).

### Generation of stable SNAP-tag cell lines

Stable HEp-2 cell lines expressing Golgi localized- or endoplasmic reticulum (ER) localized-SNAP-tag were generated by lentiviral transduction. The Golgi localized SNAP-tag cell line is based on the plasmid GalT-GFP-SNAP, encoding the first 120 amino acids of galactosyl transferase fused to EGFP and SNAP [33]. The ER localized SNAP-tag cell line is based on a plasmid encoding the ER signal sequence of hen lysozyme fused to mCherry and KDEL for ER retention. The mCherry sequence was substituted by EGFP-SNAP to produce a cell line expressing ER-GFP-SNAP-KDEL. The SNAP plasmids were subcloned into a Gateway ENTRY vector by standard molecular biology techniques. From this vector, lentiviral transfer vectors were generated by recombination with either pCDH-EF1a-GW-IRES-Bsd (a gateway-enabled derivative of pCDH-EF1a-MCS-IRES-Puro (Systems Biosciences, CA, USA)) or pCDH-PGK-GW-IRES-Puro. Lentivirus particles were packaged using a third-generation packaging system (Addgene plasmid numbers 12251, 12253 and 12259) as previously described [53, 54]. Cells were then transduced with low virus titres (multiplicity of infection (m.o.i.) < 1) and stable cell pools were generated by selection with blasticidin (3 μg/ml) or puromycin (1 μg/ml).

### Analysis of Stx transport to the Golgi and ER by the SNAP-tag method

Stx was labeled with the SNAP-tag substrate BG-GLA-NHS (New England BioLabs) according to the manufacturer's instructions and using 3:1 ratio BG to toxin. Stx1-mut-BG was then labeled with iodine as described in [13]. HEp-2 cells stably expressing GalT-GFP-SNAP or ER-GFP-SNAP were treated with 1 mM FDG for 4 h or with 2 μg/ml brefeldin A (BFA) for 30 min in complete growth medium. To analyze Golgi transport, HEp-2-GalT-GFP-SNAP cells were incubated with 50 ng/ml ^125^I-Stx1-mut-BG for 1 h in the presence of the drugs. For Stx transport to the ER, Hep-2-ER-GFP-SNAP cells were incubated with 50 ng/ml ^125^I-Stx1-mut-BG for 5 h. Then the cells were lysed, and Stx-GalT-GFP-SNAP or Stx-ER-GFP-SNAP (later referred to as Stx-SNAP) was immunoprecipitated from the lysates by GFP-trap (ChromoTek GmbH) and run on SDS-PAGE. Proteins were transferred to PVDF membranes, visualized by autoradiography and quantified using the Quantity One 1-D Analysis Software (Bio-Rad Laboratories). Both Stx subunits, A and B, were coupled to the SNAP-tag proteins, and gave essentially similar quantitative data, although the signal for ^125^I-StxB-SNAP was brighter, and thus was used for the final quantification.

### Confocal microscopy

HEp-2 cells expressing GalT-GFP-SNAP or ER-GFP-SNAP were treated with FDG for 4 h prior to incubation with Alexa555 labeled Stx1-mut-BG for 1 h (toxin labeling was performed using Alexa Fluor^®^ 555 Microscale Protein Labeling Kit, Molecular Probes^®^). To allow Stx transport to the ER, HEp-2-ER-GFP-SNAP cells were washed and incubated with fresh complete medium for additional 4 h. The cells were fixed with 10% (w/v) formalin-solution for 15 minutes, followed by permeabilization in 0.1% (w/v) Triton X-100 for 5 minutes at room temperature. The cells were incubated in blocking solution (5% (v/v) FBS in PBS) for 30 minutes, before incubation with the appropriate primary and secondary antibodies. The cells were mounted in Prolong^®^Gold containing DAPI (Molecular Probes^®^), and imaged on a laser scanning confocal microscope LSM 780 (Carl Zeiss), equipped with a 63x objective, NA 1.4. Images were prepared using ImageJ software [55].

### Lipid extraction for MS analyses

Cells were treated with or without 1 mM FDG in complete growth medium for 4 and 24 h, and then harvested for lipidomic analyses as described in [13]. Lipids were extracted from 0.5-1.5 million cells containing 150-230 μg of protein using a modified Folch lipid extraction procedure [56]. Known amounts of deuterium-labeled or heptadecanoyl-based synthetic internal standards of LPC, PC, PE, PS, PG, PA, DAG, CE, Cer, GlcCer, LacCer and Gb3, were added and used for quantification of the endogenous lipid species as described [57, 58]. Following lipid extraction, samples were reconstituted in 1:2 (v/v) chloroform:methanol and stored at −20°C prior to MS analysis of two replicates.

### MS analyses

The species of all phospholipids, SM, DAG and CE were analyzed by shotgun analysis on a hybrid triple quadrupole/linear ion trap mass spectrometer (QTRAP 5500, AB SCIEX) equipped with a robotic nanoflow ion source (NanoMate HD, Advion Biosciences) [59]. These analyses were performed using both positive and negative ion modes using multiple precursor ion scanning (MPIS) and neutral loss (NL) based methods [56, 60], whereas CEs were analyzed in positive ion mode [61]. Sphingolipids were analyzed by reverse phase ultra-high pressure liquid chromatography (UHPLC) as previously described [62] using an Acquity BEH C18, 2.1×50 mm column with a particle size of 1.7 μm (Waters, Milford) coupled to a hybrid triple quadrupole/linear ion trap mass spectrometer (QTRAP 5500, AB SCIEX). A 25 min gradient using 10 mM ammonium acetate in water with 0.1% (v/v) formic acid (mobile phase A) and 10 mM ammonium acetate in 4:3 (v/v) acetonitrile:2-propanol containing 0.1% (v/v) formic acid (mobile phase B) was used. Quantification of sphingolipids was performed using multiple reaction monitoring. Data from one such experiment are shown; similar results were obtained in another independent experiment.

### Data processing

The MS data files were processed as previously described [57] using Lipid Profiler™ and MultiQuant™ software. Lipids were normalized to their respective internal standard [57] and the concentrations of molecular lipids are presented as nmol/mg protein. Quality control samples were utilized to monitor the overall quality of the lipid extraction and mass spectrometry analyses [58] mainly to remove technical outliers and lipid species that were detected below the limit of quantification.

For clarity, minor species constituting less than 1% of the whole class for Cer, GlcCer, LacCer and Gb3, less than 5% for PI and DAG, and less than 2% for the rest of the lipid classes are not shown in the figures. The data are presented either as nmol of lipid per mg of protein (to show absolute values of the Gb3 and its precursors), or as mol% of all lipids (for the summary of lipid classes) or as mol% of the total class (for data on species composition of a single lipid class). The relative fold increase/decrease was calculated as a fold change between the mol% of the lipid in FDG-treated compared to control samples.

### Annotations of lipid species

The different lipid species of phosphatidylcholine (PC), phosphatidylethanolamine (PE), phosphatidylserine (PS), phosphatidylinositol (PI), phosphatidic acid (PA), phosphatidylglycerol (PG) and diacylglycerol (DAG) are listed with the two fatty acyl groups separated with an underscore (*sn*-position of the fatty acids is not known) or with a slash (for identical fatty acyl groups), e.g. PC 16:0_18:1 and PC 16:0/16:0, according to Liebisch and colleagues [63]. LysoPC and lysoPE are abbreviated as LPC and LPE, respectively, and cholesteryl esters are abbreviated CE. The ether-linked phospholipids are shown as PC O (alkyl), PC P (alkenyl), PE O (alkyl) and PE P (alkenyl). The N-amidated fatty acyl groups for sphingomyelin (SM), ceramide (Cer) and glycosphingolipids are shown after the slash, e.g. SM d18:1/16:0. Abbreviations for glycosphingolipids are: glucosylceramide (GlcCer), lactosylceramide (LacCer) and globotriaosylceramide (Gb3).

## SUPPLEMENTARY DATA




