# Supplementary material for: Cellular effects of fluorodeoxyglucose: Global changes in the lipidome and alteration in intracellular transport
Source: Oncotarget. 2016 Nov 4;7(48):79885–900. doi: 10.18632/oncotarget.13089 (PMC5346758; doi:10.18632/oncotarget.13089)
Supplement: Supplementary file 1 [file oncotarget-07-79885-s001.pdf]

Cellular effects of fluorodeoxyglucose: Global changes in the lipidome and alteration in intracellular transport

SUPPLEMENTARY DATA

SUPPLEMENTARY MATERIALS AND METHODS

Endocytosis of Shiga toxin

Stx1-mut was biotinylated with the reducible EZ-Link Sulfo-NHS-SS-Biotin (Pierce Biotechnology) followed by the labeling with ^{125}I (^{125}I-Stx1-mut-biotin). After treatment with FDG, HEp-2 cells were incubated with 40 ng/ml ^{125}I-Stx1-mut-biotin for 20 min at 37°C in HEPES buffered medium supplemented with 100 µg/ml BSA. This incubation time is too short for the toxin to reach the ER and its reducing environment. The cells were washed with cold buffer (0.14 M NaCl, 2 mM $CaCl_2$, 20 mM HEPES, pH 8.6), and then half of the cells were treated with 0.1 M sodium 2-mercaptoethanesulfonate (MESNa) in the same buffer for 30 min on ice. MESNa reduces the SS-biotin from the cell surface-bound toxin and thereby allows us to determine the amount of internalized toxin. The other half of the cells were mock-treated to determine the amount of total cell-associated toxin (internalized + cell surface-bound toxin). All cells were then washed and lysed in a 100 µl/well lysis buffer (0.1 M NaCl, 5 mM $MgCl_2$, 1% (v/v) Triton X-100, 20 mM HEPES, 60 mM *n*-octyl-β-pyranoside, pH 7.4). The lysates were mixed with 20 µg streptavidin-coated Dynabeads (Life Technologies) diluted in PBS supplemented with 0.2% (w/v) BSA and 0.5% (v/v) Tween-20, and incubated for 1 h at room temperature under rotation. The captured ^{25}I-Stx1-mut-biotin was counted by a γ-counter (1261 Multigamma Gamma Counter, Wallac). Endocytosis of Stx was calculated as internalized toxin (remaining signal after MESNa treatment) in percentage of total cell-associated toxin (mock treated cells).

To measure the background signal for the endocytosis, cells were pre-cooled before the addition of the ^{125}I-Stx1-mut-biotin, and the incubation with the toxin was performed on ice. Further steps were identical to those described above. As Stx binds to the cells but is not taken up when added on ice, all biotin is expected to be removed from the toxin upon MESNa treatment. In all experiments the remaining background signal after MESNa treatment was lower than 10% of total Stx signal. The background values were subtracted from the measured Stx endocytosis values.

Quantitative real-time reverse transcription (qRT)-PCR

After the treatment, HEp-2 cells were washed twice with sterile ice-cold PBS and then stored in a -80°C freezer. Total RNA was isolated using RNeasy Plus Mini Kit and QIAcube (QIAGEN) for automated sample handling. For RNA purification, the protocol "Purification of total RNA from easy-to-lyse animal tissues and cells using gDNA Eliminator columns" (version 2, October 2007, QIAGEN) was used. If not analyzed immediately, samples with isolated RNA were stored in a -80°C freezer. The RNA concentration in the samples was measured with NanoDrop 2000 spectrophotometer (Thermo Scientific). Complementary DNA (cDNA) was synthesized from 0.5 µg/sample of the isolated RNA using iScript cDNA synthesis kit (Bio-Rad Laboratories). As a negative control, one additional tube was prepared containing all components except for the reverse transcriptase. qRT-PCR was performed using LightCycler 480 SYBR Green I Master kit and LightCycler 480 (Roche Diagnostics). All samples were run in duplicates, and serial dilutions of one cDNA sample were used for standard curve plotting. All primers were QuantiTect Primer Assays (QIAGEN): Gb3 synthase (*Gb3s*) (Hs_A4GALT_1_SG), GlcCer synthase (*GCS*) (Hs_UGCG_1_SG), and TATA-binding protein (*TBP*) (Hs_TBP_1_SG). Relative quantification of the ratio between target (*Gb3s* or *GCS*) and reference (*TBP*) genes was performed using LightCycler 480 Analysis software.

Analysis of Stx binding by flow cytometry

Stx1-mut was labeled using the AlexaFluor-555 Microscale Labeling Kit (Thermo Fisher Scientific) according to the manufacturer's recommendations. In brief, the buffer of Stx1-mut (at 0.25 µg/µl) was exchanged from TRIS-buffer to PBS using Amicon Ultra-0.5 Centrifuge Filter devices (Millipore). Then 20 µg Stx1-mut was mixed with 1/10 volume of 1 M sodium bicarbonate and 0.6 µl of AlexaFluor555 dye and incubated at room temperature for 15 min. The labeled protein was separated from the unreacted dye by filtering through a gel resin settled in a spin column, and centrifuged at 16 000 *g* for 1 min. The concentration of the labeled protein was determined using NanoDrop 2000 (Thermo scientific) and stored in small aliquots at -20°C.

Cells were seeded in 6 well culture plates one day before treatment with 1 mM FDG for 24 h or 72 h in complete growth medium. Then the cells were trypsinized, spun down at 1000 rpm for 5 min, and resuspended in cold PBS supplemented with 2% (v/v) fetal bovine serum (FBS) (PBS/FBS). Each sample was divided into two sets and one set of the sample was left unstained (negative), while the other was incubated with 200 ng/ml Stx1-mut-Alexa555 in PBS/FBS for 30 min on ice. Thereafter, the cells were spun down (1000 rpm, 5 min) and washed twice in PBS/FBS before being run on LSR II flow cytometer (BD Biosciences) for data acquisition. Cell debris and cell aggregates were gated out according to forward and side scattering, and the AlexaFluor555 signal was quantified only for a single cell population. The gating, data analysis and visualization were performed using Cytobank (www.cytobank.org).

Glycosphingolipid extraction and analysis by HPTLC

Cells were seeded in 225 cm^2 culture flasks one day prior to treatment with 1 mM FDG for 24 h or 72 h. Cells were harvested by trypsinization and counted using Countess II Automated Cell Counter (Life Technologies), and 1-2 · 10^7 cells/sample were transferred to methanol pre-washed centrifuge glass tubes. The cells were centrifuged at 400 *g* for 8 min at 10°C, and the pellet was resuspended in 10 ml methanol. The suspension was then sonicated for 15 min and centrifuged at 5500 *g* for 10 min at 10ºC. The supernatant was transferred to a 60 ml SampleGenie Genevac glass bottles by means of filtration. The pellet was resuspended in 10 ml 1:2 (v/v) chloroform:methanol following the sonication and centrifugation steps as described above. The procedure was repeated two more times resuspending the pellet in 1:1 (v/v) and 2:1 (v/v) chloroform:methanol, and the supernatants were collected in Genevac glass bottle. The samples were dried using Genevac EZ-2 automated evaporation system (Genevac).

For hydrolysis of esterified lipids, dried lipid extracts were incubated with 4.5 ml 1M NaOH for 1 h at 37ºC. Then 450 µl 10 M HCl was added, and the samples were transferred to dialysis tubes (Spectra/Pro No4, MWCO 12 000-14 000) followed by the dialysis against water for 2 days. The extracts were then dried using Genevac EZ-2 automated evaporation system (Genevac) and resuspended in 2:1 (v/v) chloroform:methanol with a volume of 12.5 µl per one million cells.

The extracts were applied to HPTLC Silica gel 60 plates using Linomat 5 (CAMAG) and chromatographed with a mixture of chloroform:methanol:water (70:30:4) as a mobile phase, followed by staining with 0.3% (w/v) orcinol solution in 3M H_2SO. Developed plates were scanned using GS-800 Calibrated Densitometer (Bio-Rad Laboratories) and the bands were quantified by Quantity One 1-D Analysis Software (Bio-Rad Laboratories).

Calcium-release assay

Changes in intracellular calcium were measured using the Fluo-4 NW calcium Assay Kit from Molecular probes (Oregon) using HEPES buffered calcium-free Hank's balanced salt solution (Life Technologies) for cell loading with the Fluo-4 dye and during the fluorescence measurements. HEp-2 cells were seeded in 96-well plates at a density of 1×10^4 cells/well one day before the experiment. The cells were incubated with 1 mM FDG or 10 mM 2DG in HEPES buffered medium for 3 h, and then loaded with the Fluo-4 dye for 1 h in presence of the drugs. For rapid Ca^{2+} release from the ER, 1 µM thapsigargin (TG) was automatically added using a syringe dispenser. The fluorescence was measured every 20 sec, 3 min before and 10 min after the addition of TG, on a Synergy2 plate reader (BioTek) using 485/20 and 528/20 excitation and emission filters, respectively. Four replicate wells were measured at each time point, and the results are presented as change in fluorescence over time relative to the average pre-injection fluorescence.

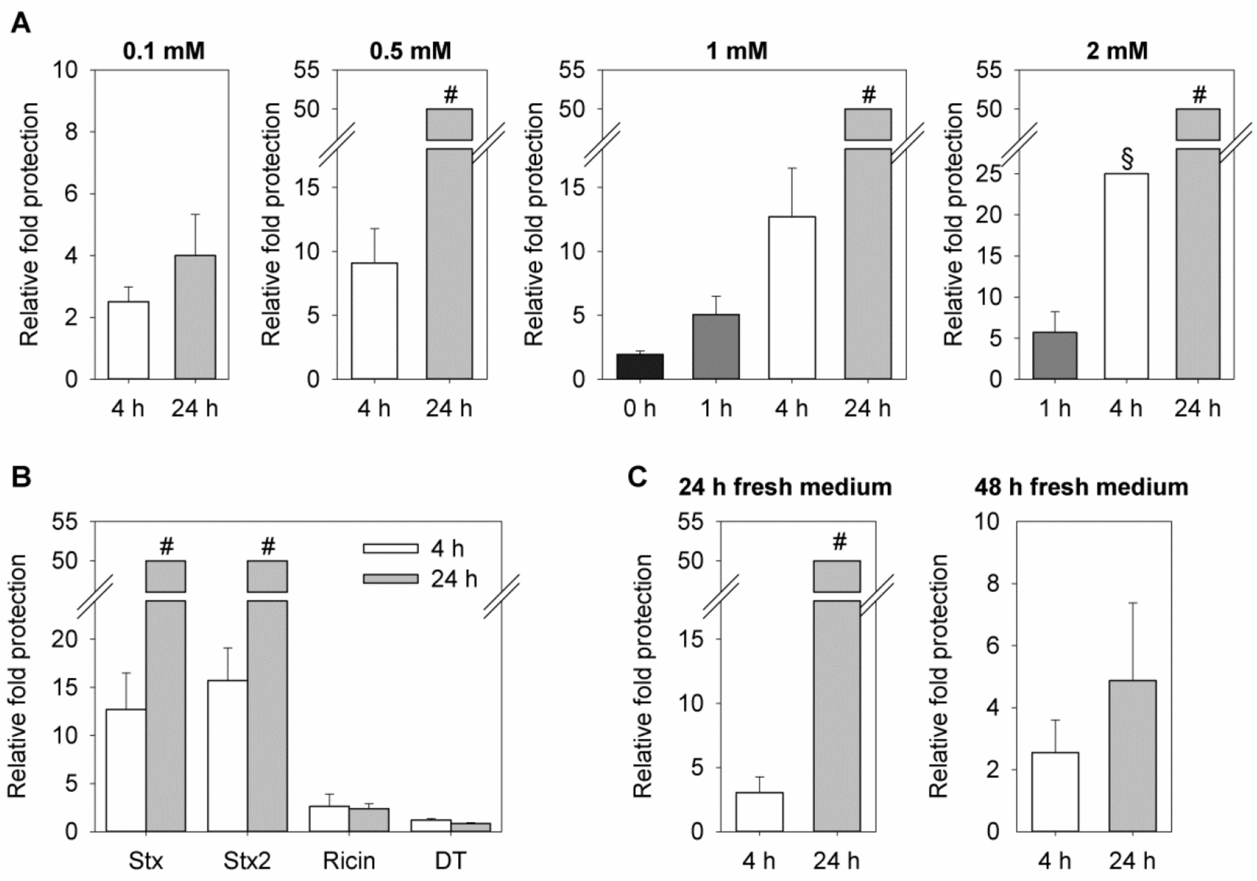

Supplementary Figure S1: HEp-2 cell protection against Shiga toxins, ricin and diphtheria toxin by FDG. A. HEp-2 cells were treated with 0.1 mM, 0.5 mM, 1 mM or 2 mM FDG prior to 3 h incubation with ten-fold serial dilutions of Shiga toxin (Stx) in leucine-free medium in the presence of FDG. Cells were then incubated in the presence of [^{3}H]leucine for 20 min, and protein synthesis was measured. Relative fold protection was calculated as an increase in the concentration of the toxin required to inhibit protein synthesis by 50%. The graphs show mean protection against Stx for FDG concentrations 0.1, 0.5, 1 and 2 mM; the error bars show the deviation from the mean of two independent experiments for FDG concentrations 0.1 mM, 0.5mM and 2 mM, and the error bars show SEM (n=4) for 1 mM FDG. The error bar for 4 h treatment with 2 mM FDG could not be calculated (marked as §), because in one of the two independent experiments the protection was higher than 50 fold, while in the other it was 25 fold. **B.** The graph shows mean protection against Stx, Shiga-like toxin 2 (Stx2), ricin and diphtheria toxin (DT) after cell pre-treatment with 1 mM FDG for 4 h or 24 h (experiment performed as described in (A), but subjecting cells to different toxins); the error bars show the SEM for Stx (n=4) or deviation from the mean of two independent experiments for Stx2, ricin and DT. **C.** Following 4 h or 24 h treatment with 1 mM FDG, HEp-2 cells were washed twice with medium and grown in complete growth medium without FDG for 24 h or 48 h prior to measuring protection against Stx as described in (A). The graphs show mean protection against Stx; the error bars show the deviation from the mean of two independent experiments. (A-C) In the samples where the highest toxin concentration tested (100 ng/ml) did not reduce protein synthesis down to 50%, the fold-protection could not be calculated and was considered to be more than 50-fold (marked as #).

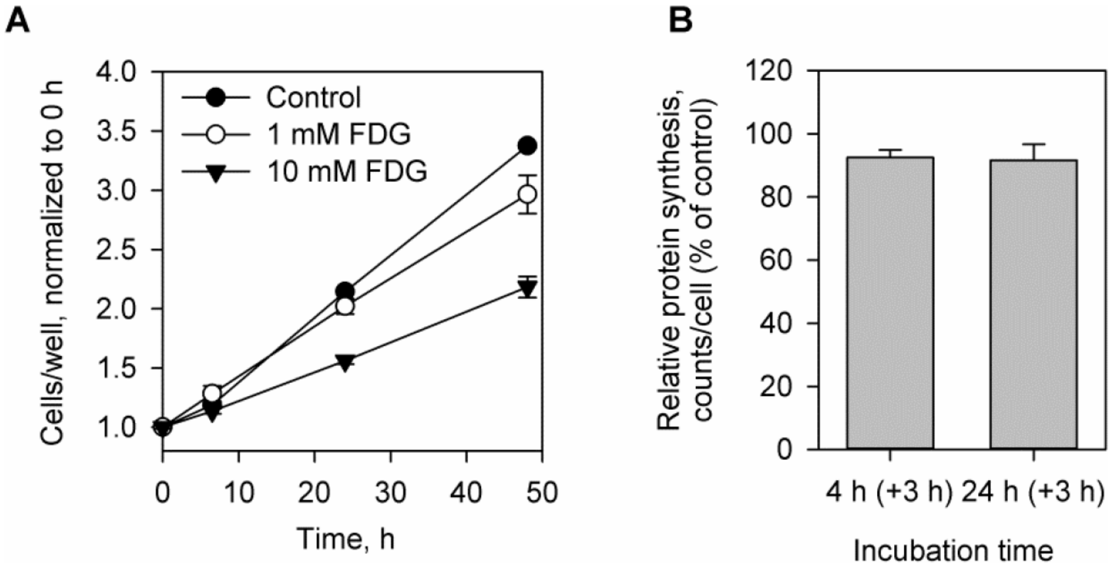

Supplementary Figure S2: Effect of FDG on growth and protein synthesis in HEp-2 cells. A. HEp-2 cells were grown in complete DMEM growth medium with or without FDG and counted. The graph shows cell number per well normalized to time point 0 h; the error bars show the deviation from the mean of two independent experiments. **B.** HEp-2 cells were treated with 1 mM FDG for 4 or 24 h in complete DMEM growth medium. Then, the medium was changed to leucine-free HEPES buffered medium with or without 1 mM FDG, and the cells were incubated for additional 3 h prior to addition of [^{3}H]leucine for 20 min. The rate of protein synthesis was measured as incorporation of [^{3}H]leucine into newly synthesized proteins, and plotted as a percentage of control samples +SEM, n=5.

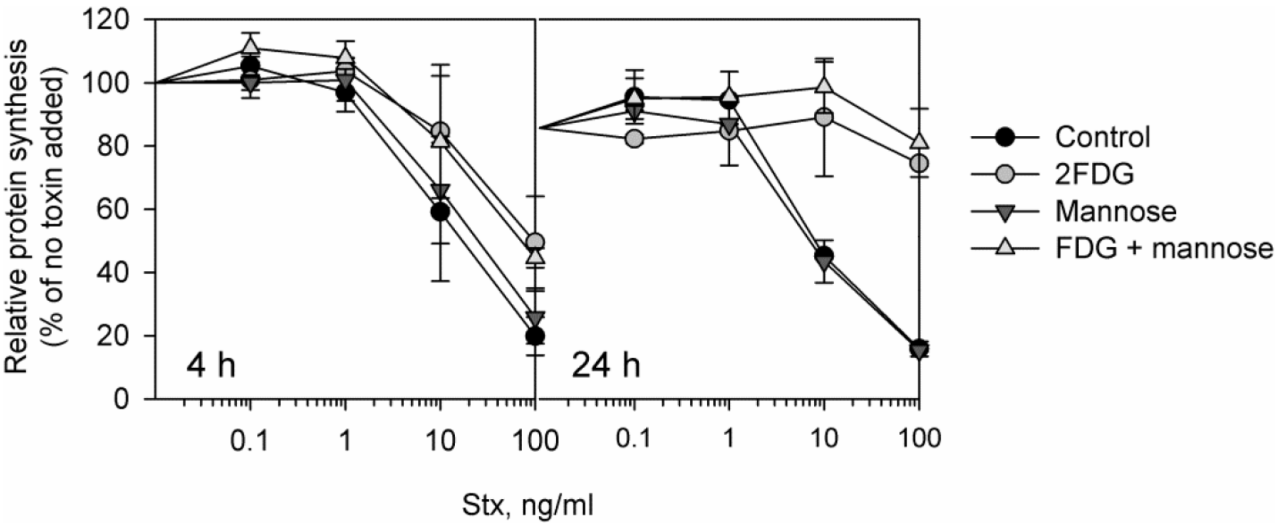

Supplementary Figure S3: Mannose does not rescue cell sensitivity to Stx. HEp-2 cells were treated with 1 mM FDG, 4 mM mannose or both for 4 or 24 h in complete growth medium, followed by the incubation with ten-fold serial dilutions of Shiga toxin (Stx) for 3 hours in leucine-free medium with or without the drugs. The cells were then incubated in the presence of [^{3}H]leucine for 20 min, and protein synthesis was measured. The graph shows mean values of two independent experiments; the error bars represent the deviation from the mean.

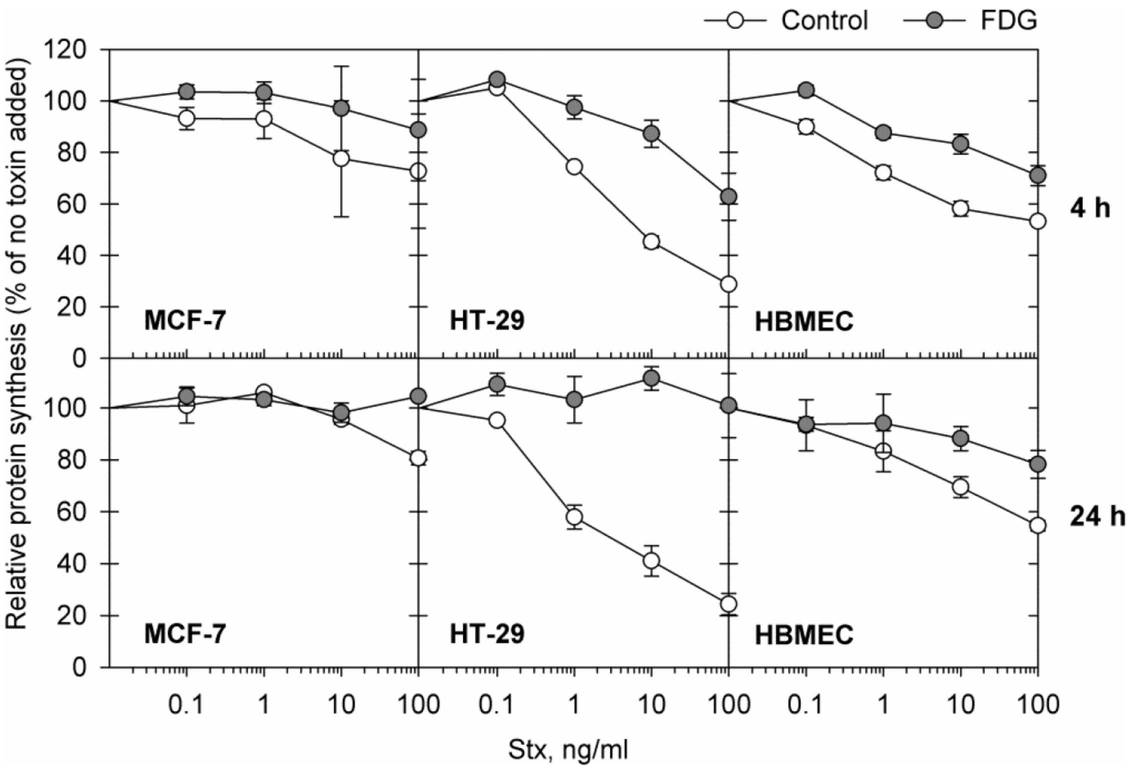

Supplementary Figure S4: FDG protects MCF-7, HT-29 and HBMEC cells against Stx. Cells were treated with or without 1 mM FDG for 4 or 24 h in complete growth medium, followed by the incubation with ten-fold serial dilutions of Stx for 3 hours in leucine-free medium with or without FDG. The cells were then incubated in the presence of [^{3}H]leucine for 20 min, and protein synthesis was measured. The graph shows mean values of two independent experiments for MCF-7 and HT-29 cells (the error bars represent the deviation from the mean), and mean values of three independent experiments for HBMEC cells (±SEM).

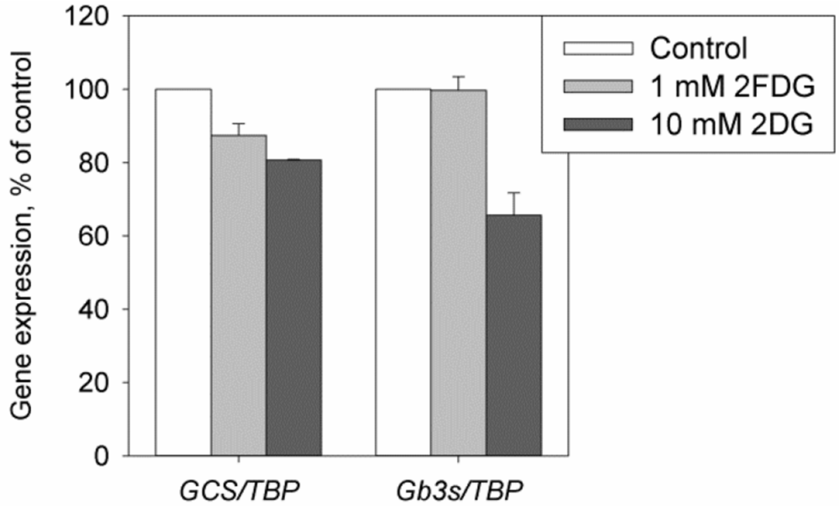

Supplementary Figure S5: Expression of the GlcCer synthase gene (*GCS*) and the Gb3 synthase gene (*Gb3s*) following FDG and 2DG treatment. HEp-2 cells were treated with 1 mM FDG or 10 mM 2DG for 6 h prior to total RNA isolation and qRT-PCR analysis. The graph shows mean expression levels of the *GCS* and *Gb3s* (*TBP* was used as a reference for quantification); the error bars show the deviation from the mean of two independent experiments.

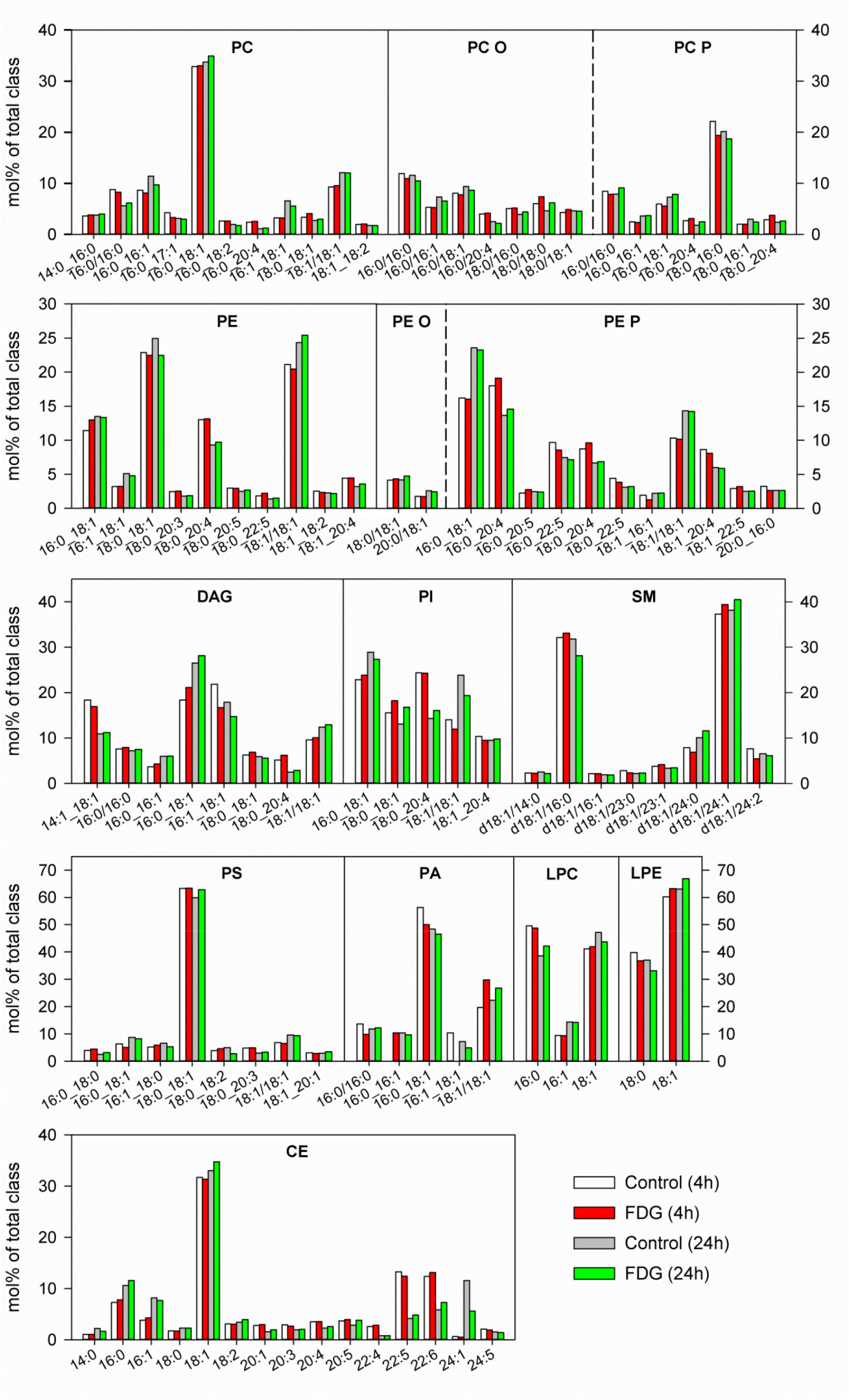

Supplementary Figure S6: Species composition of major lipid classes HEp-2 cells were treated with or without 1 mM FDG for 4 h or 24 h, and whole cell lysates were analyzed by MS. The amount of individual lipid species was quantified as percentage of the total content of the lipid class. For clarity, minor species are not presented in the graph (for full species list and quantitative data see Online Supplementary Material 2). It should be noted that the PC P and PE P species representation might contain an overlapping contribution from the isobaric alkyl species of the respective class (PC O and PE O), which could not be determined by the applied methodology.

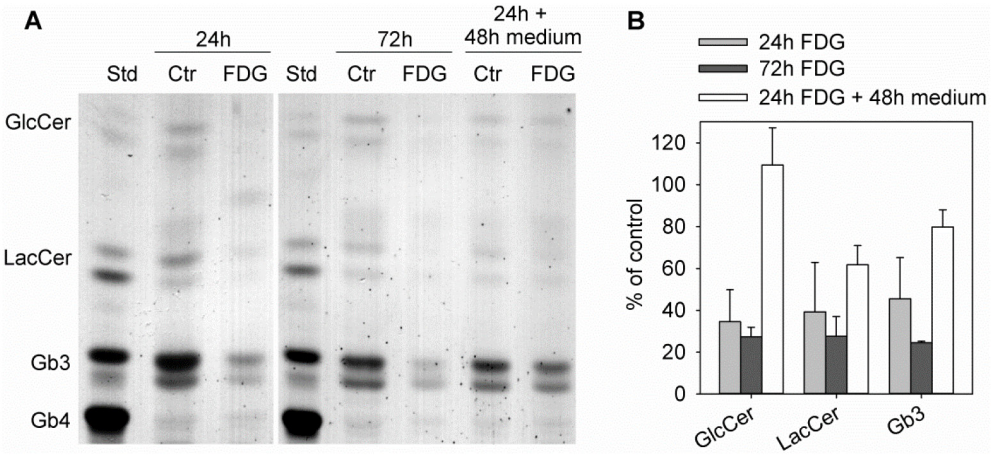

Supplementary Figure S7: Glycosphingolipid levels are partially restored 48 h after FDG removal. HEp-2 cells were treated with or without 1 mM FDG for 24 h or 72 h, or following 24 h treatment with FDG the cells were washed and incubated with complete growth medium without the drug for 48 h. Cellular glycosphingolipids were extracted with chloroform/methanol as described in the Supplementary Methods, and the extracts were applied on a silica plate, chromatographed and visualised by orcinol staining. **A.** Representative chromatograms. **B.** Quantification of total GlcCer, LacCer and Gb3 content in the cells. Since GlcCer, LacCer and Gb3 each separates into two bands, which represent lipid species with different length of the fatty acyl chains, both bands were quantified. The total lipid class represents the sum of the two bands. The graph shows relative cellular levels of GlcCer, LacCer and Gb3 compared to control (untreated) cells; the error bars show the deviation from the mean of two independent experiments.

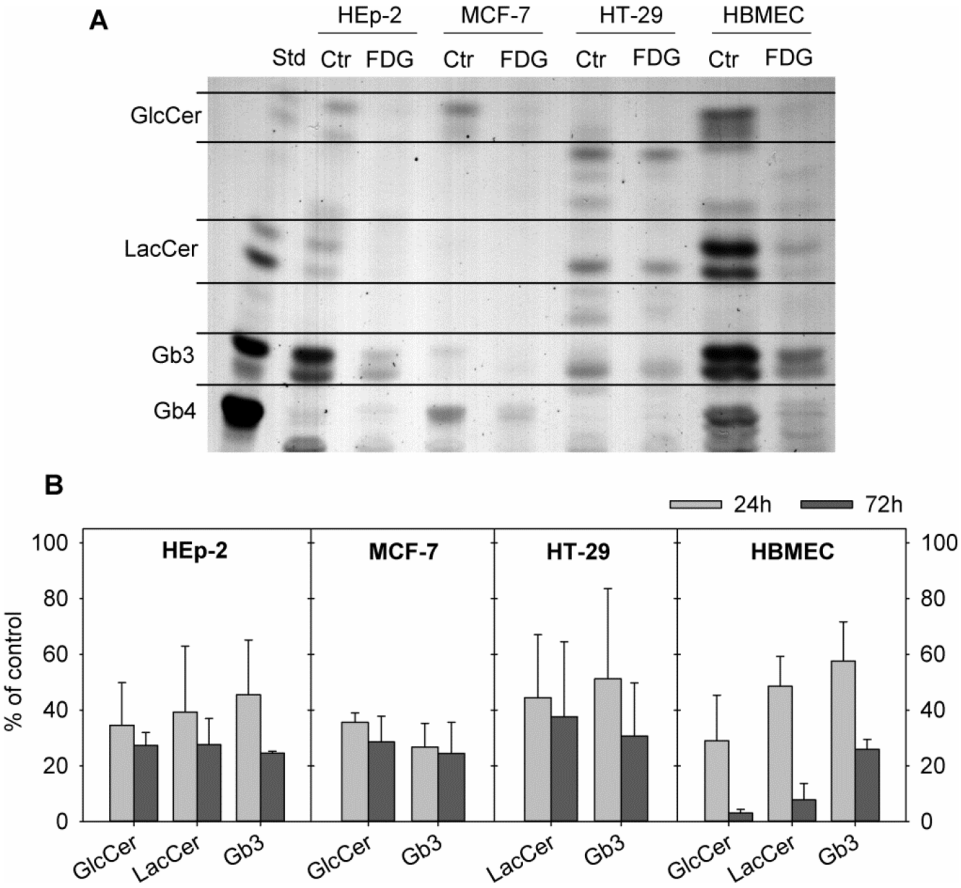

Supplementary Figure S8: FDG treatment reduces cellular glycosphingolipid levels in HEp-2, MCF-7, HT-29 and HBMEC cells. HEp-2, MCF-7, HT-29 and HBMEC cells were treated with or without 1 mM FDG for 24 h or 72 h prior to glycosphingolipid extraction with chloroform/methanol as described in the Supplementary Methods. The extracts were applied on a silica plate, chromatographed and visualised by orcinol staining. **A.** Representative chromatogram for 72 h treatment with FDG. Several classes of glycosphingolipids separate into two bands, because very long-chain species move longer (higher up) in the chromatogram than the long-chain species. Thus, the added horizontal lines indicate the bands which were quantified for GlcCer, LacCer and Gb3 **B.** Quantification of total GlcCer, LacCer and Gb3 content in the cells. When two bands were present, both bands were quantified and the total lipid class represents the sum of the two bands. The graph shows relative cellular levels of GlcCer, LacCer and Gb3 compared to control (untreated) cells; the error bars show the deviation from the mean of two independent experiments.

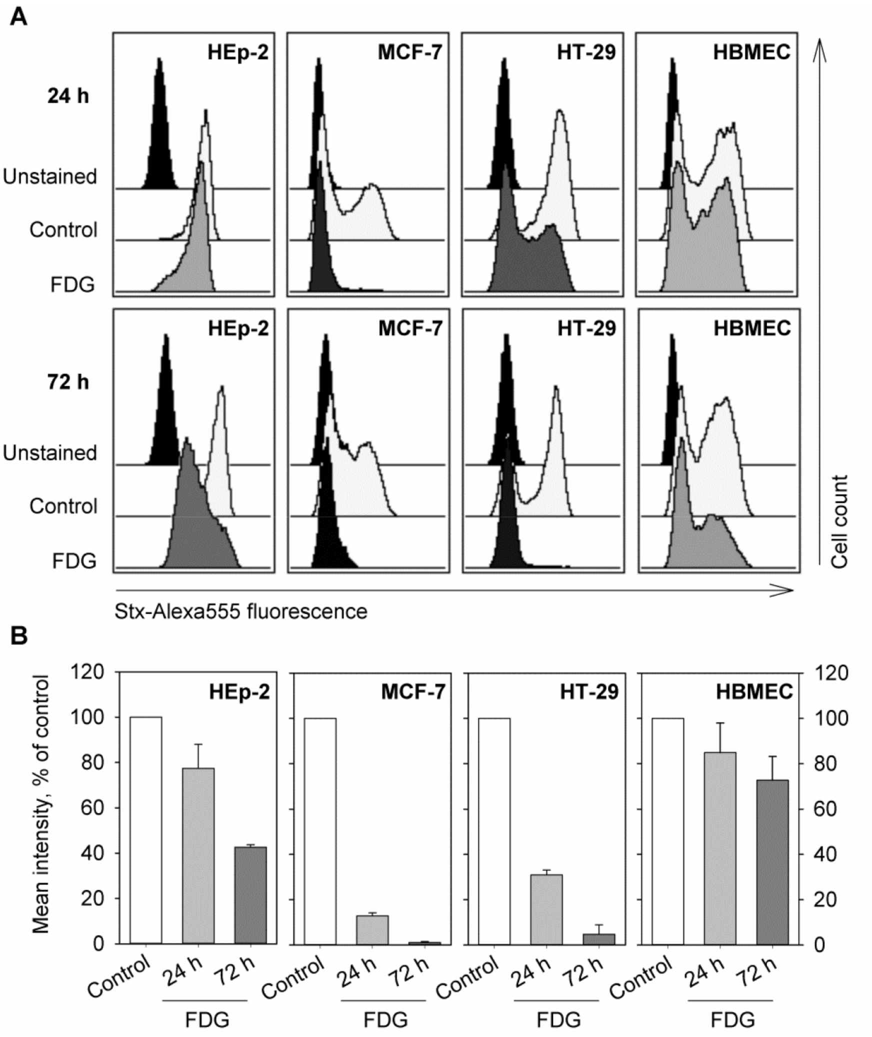

Supplementary Figure S9: FDG reduces Stx binding to HEp-2, MCF-7, HT-29 and HBMEC cells. Cells were treated with 1 mM FDG in complete growth medium for 24 h or 72 h, trypsinized and then incubated with 200 ng/ml Stx1-mut-Alexa555 for 30 min on ice. The data were acquired by LSR II flow cytometer (BD Biosciences), and the gating, data analysis and visualization were performed using Cytobank (www.cytobank.org). **A.** The cell count is plotted as a function of the fluorescence intensity (Stx-Alexa555) with grey coding representing the mean intensity of the signal; unstained – control cells not incubated with Stx1-mut-Alexa555; the graph shows one of two independent experiments. **B.** Mean intensity of Stx-Alexa555 signal per cell was quantified and plotted as a percentage of the control; the error bars show the deviation from the mean of two independent experiments.

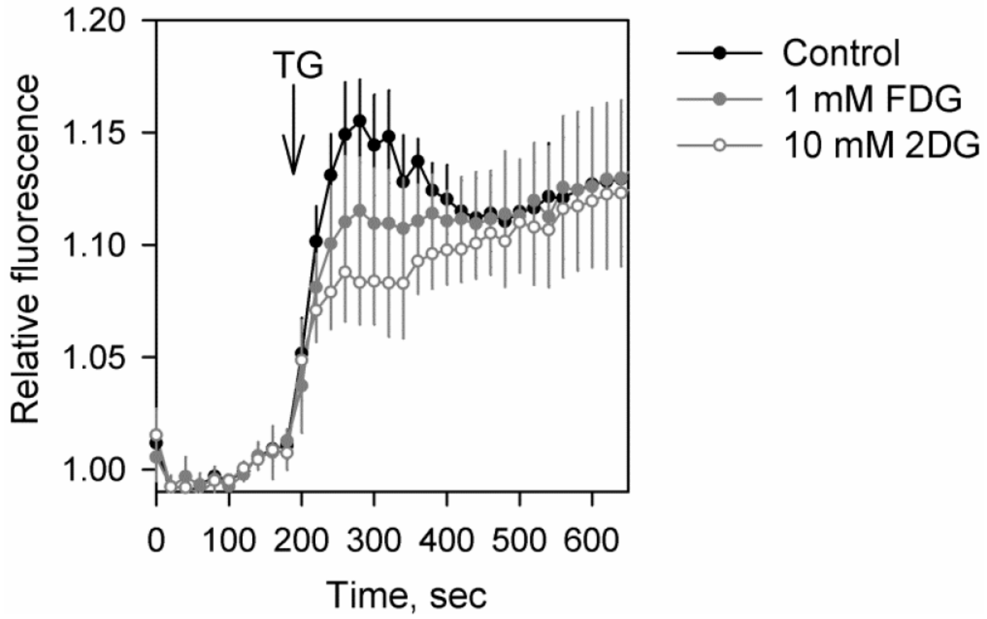

Supplementary Figure S10: FDG leads to partial depletion of ER calcium reservoirs. HEp-2 cells were treated with 1 mM FDG or 10 mM 2DG for 3 h and then loaded with Fluo-4 dye for 1 h in presence of the drugs. TG was automatically added 3 min after the start of fluorescence measurements. The results are presented as change in fluorescence over time relative to average pre-injection fluorescence, and the graph shows mean values from three independent experiments (±SEM).

Supplementary Table S1: Lipid Concentrations Per Sample

See Supplementary File 1
